# A Multi-Pollutant Air Quality Health Index (AQHI) Based on Short-Term Respiratory Effects in Stockholm, Sweden

**DOI:** 10.3390/ijerph16010105

**Published:** 2019-01-02

**Authors:** Henrik Olstrup, Christer Johansson, Bertil Forsberg, Andreas Tornevi, Agneta Ekebom, Kadri Meister

**Affiliations:** 1 Atmospheric Science Unit, Department of Environmental Science and Analytical Chemistry, Stockholm University, 11418 Stockholm, Sweden; christer.johansson@aces.su.se; 2 Environment and Health Administration, SLB, Box 8136, 104 20 Stockholm, Sweden; 3 Division of Occupational and Environmental Medicine, Department of Public Health and Clinical Medicine, Umeå University, 90187 Umeå, Sweden; bertil.forsberg@umu.se (B.F.); andreas.tornevi@umu.se (A.T.); 4 Swedish Museum of Natural History, 114 18 Stockholm, Sweden; agneta.ekebom@nrm.se; 5 Umeå School of Business, Economics and Statistics (USBE), Umeå University, 90187 Umeå, Sweden; kadri.meister@umu.se

**Keywords:** AQHI, asthma, NO_x_, ozone, PM_10_, birch pollen, risk coefficients

## Abstract

In this study, an Air Quality Health Index (AQHI) for Stockholm is introduced as a tool to capture the combined effects associated with multi-pollutant exposure. Public information regarding the expected health risks associated with current or forecasted concentrations of pollutants and pollen can be very useful for sensitive persons when planning their outdoor activities. For interventions, it can also be important to know the contribution from pollen and the specific air pollutants, judged to cause the risk. The AQHI is based on an epidemiological analysis of asthma emergency department visits (AEDV) and urban background concentrations of NO_x_, O_3_, PM_10_ and birch pollen in Stockholm during 2001–2005. This analysis showed per 10 µg·m^–3^ increase in the mean of same day and yesterday an increase in AEDV of 0.5% (95% CI: −1.2–2.2), 0.3% (95% CI: −1.4–2.0) and 2.5% (95% CI: 0.3–4.8) for NO_x_, O_3_ and PM_10_, respectively. For birch pollen, the AEDV increased with 0.26% (95% CI: 0.18–0.34) for 10 pollen grains·m^–3^. In comparison with the coefficients in a meta-analysis, the mean values of the coefficients obtained in Stockholm are smaller. The mean value of the risk increase associated with PM_10_ is somewhat smaller than the mean value of the meta-coefficient, while for O_3_, it is less than one fifth of the meta-coefficient. We have not found any meta-coefficient using NO_x_ as an indicator of AEDV, but compared to the mean value associated with NO_2_, our value of NO_x_ is less than half as large. The AQHI is expressed as the predicted percentage increase in AEDV without any threshold level. When comparing the relative contribution of each pollutant to the total AQHI, based on monthly averages concentrations during the period 2015–2017, there is a tangible pattern. The AQHI increase associated with NO_x_ exhibits a relatively even distribution throughout the year, but with a clear decrease during the summer months due to less traffic. O_3_ contributes to an increase in AQHI during the spring. For PM_10_, there is a significant increase during early spring associated with increased suspension of road dust. For birch pollen, there is a remarkable peak during the late spring and early summer during the flowering period. Based on monthly averages, the total AQHI during 2015–2017 varies between 4 and 9%, but with a peak value of almost 16% during the birch pollen season in the spring 2016. Based on daily mean values, the most important risk contribution during the study period is from PM_10_ with 3.1%, followed by O_3_ with 2.0%.

## 1. Introduction

The air quality index (AQI) is a numerical scale that has been used in order to describe the air quality situation in relation to a reference value at a given time and place. An AQI is usually based on several pollutants, where each pollutant is compared with its air quality limit value, and the pollutant with the highest value in relation to its limit value determines the AQI-value [1]. In this case, one individual pollutant determines the index, but without considering the other pollutants with smaller values in relation to their limit values, which means that the cumulative effect is not captured. Another limitation is that the AQI does not capture the no-threshold concentration-response relationship between air pollution exposure and health [2]. The AQI is usually based on pollutant concentrations at a few monitoring stations, often located at hot-spots, but without reflecting the spatial variations in the city, and consequently not the health risks for the general populations associated with air pollution exposure.

The air quality health index (AQHI) is a tool that has been developed in order to reflect the health risks associated with simultaneous exposure to several different air pollutants. In a number of cities in Canada, the AQHI is based on the sum of the mortality risks associated with short-term exposure to different air pollutants. Different combinations of CO, SO_2_, NO_2_, O_3_, PM_2.5_ and PM_10_ are included in the AQHI, which is presented on an arbitrary scale of 0–10 [2]. A similar approach is used in Guangzhou in China, based on time-series studies of the associations between exposure to SO_2_, NO_2_, O_3_ and PM_2.5_ and mortality during the period 2012–2015. The excess mortalities associated with these pollutants are then added in order to construct an AQHI on an arbitrary scale [3]. The AQHI can be applied in different contexts. One application is to present the current air quality situation on a map as an index based on the coexistence of several different pollutants. Another application is for forecasting purposes to provide warnings regarding the air quality situation tomorrow or the next few days considering the risk as a combined effect of several pollutants.

Cairncross et al. [4] defined an Aggregate Risk Index (ARI; similar to AQHI in this paper) to describe the health effects associated with the coexistence of several different air pollutants in the city of Cape Town, South Africa. Daily mortality associated with combined short-term exposure to PM_10_, PM_2.5_, SO_2_, O_3_, NO_2_ and CO is calculated, and the total incremental daily mortality risk associated with exposure to these pollutants is scaled to an index value ranging from 0 to 10 [4].

The AQHI concept has also been evaluated by studying the associations between the AQHI value and some health outcomes on a daily basis. In Chen et al. [5], the associations between AQHI and emergency department visits for acute ischemic stroke, based on logistic regression, were investigated during the period 1998–2002 in the city of Edmonton in Alberta, Canada. The AQHI was based on mortality risks associated with a combination of NO_2_, O_3_ and PM_2.5_. A time-stratified case-crossover analysis, adjusted for temperature and humidity, was performed. The AQHI was significantly associated with emergency department visits for acute ischemic stroke during April to September, and with the strongest association in the age group 75 years and above.

In Kousha et al. [6], a time-stratified case-crossover analysis regarding emergency department visits for urticaria was performed during the period 2004–2010 in the city of Windsor in Ontario, Canada. The AQHI was developed based on the mortality risks associated with exposure to NO_2_, O_3_ and PM_2.5_. The correlation between this AQHI and emergency department visits for urticaria was calculated by using logistic regression. Odds ratios for the association between one unit AQHI increase and emergency department visits for urticaria were presented. Significant odds ratios (95% CI) were observed for lags of 2 and 3 days.

Szyszkowicz et al. [7] performed an analysis with the same methodology as the above mentioned studies (including NO_2_, O_3_ and PM_2.5_ in the AQHI), but where the association between one unit increase in AQHI and emergency department visits for asthma was calculated in the city of Windsor in Ontario, Canada during the period 2004–2010. Significant associations between the AQHI levels and emergency department visits for asthma were observed, with the largest value lagged by 9 days in the warm season during April to September.

In To et al. [8], asthma attributed hospitalizations, emergency department visits and outpatient visits, associated with one unit increase in AQHI based on NO_x_, O_3_ and PM_2.5_, were analyzed in Ontario, Canada during the period 2003–2006. Poisson regression was used to estimate the risk ratio for the AQHI, and where adjustments for a number of possible confounders were performed. Statistically significant associations were found for one unit increase in AQHI and asthma-related hospitalizations on the same day (lag 0), and for emergency department visits after two days (lag 2).

In another paper by To et al. [9], the associations between air pollutant concentrations and emergency department visits and hospitalizations due to 11 major chronic diseases were quantified in Ontario, Canada during the period 2003–2010. The AQHI was based on NO_x_, O_3_, and PM_2.5_. The short-term effects associated with an increase in AQHI were estimated with Poisson regression, adjusting for possible confounders. One unit increase in the 10-point AQHI-scale corresponded to increases in the outpatient visits ranging from 1 to 5%.

In summary, an AQHI can be based on the cumulative effects of several different air pollutants and include different types of health outcomes like daily mortality and emergency department visits for acute ischemic stroke, asthma, urticaria or other different chronic diseases. Since the health impact is different depending on pollutant and health outcome, the AQHI will be different.

Normally, synergistic effects of air pollutants are not considered, even though such effects may well exist [10]. In addition, subpopulations may be more sensitive due to other factors such as pollen. Some studies have shown that short-term exposure to high levels of air pollutants may enhance the asthmatic response to allergens [11], but this is seldom described in the epidemiological studies. However, Guilbert et al. [12] studied the Brussels Capital Region over 6 years and reported that the daily number of hospitalizations for asthma increased by 3.2% (95% CI: 1.1–5.3) for an interquartile range increase (40 pollen grains·m^–3^) in the birch pollen concentration. There was no confounding from pollutants, but an interaction with stronger effects of birch pollen at ozone concentrations above the 85th percentile. Ito et al. [13] found an association between birch pollen concentrations and emergency department visits in New York City. Statistically significant rate ratios for no exposure vs the 98th percentile (680 pollen grains·m^–3^) were found for lag 1 and lag 2.

Which air pollutants and health outcomes to consider may therefore be critical for the AQHI that is reported to the citizens. The purpose of this study is to construct an AQHI for Stockholm based on the effects on asthma emergency department visits (AEDV) associated with combined exposure to several different air pollutants, and also to evaluate the relative importance of each pollutant during different parts of the year. We also discuss the implication for the use of an AQHI as a tool to inform the general public about the air quality situation in a city.

## 2. Materials and Methods

The air quality health index in Stockholm has been calculated based on the city-specific beta-coefficients for AEDV associated with short-term exposure to NO_x_, O_3_, PM_10_ and birch pollen according to the following equation:

(1) $$\mathrm{AQHI}=\sum_{i=1\ldots p}100\;\left(e^{\beta iXi}-1\right)$$

The beta-coefficient (β_i_) represents the increase in AEDV per 1 µg·m^–3^ increase of each individual air pollutant (*X_i_*), except for birch pollen, which is the increase of one pollen grain per cubic meter. AQHI is the total percent increase in visits due to pollutants and birch pollen (*p*). The beta-coefficients are based on all age groups, and the exposure window is lag01, which means the concentration mean of the same day and yesterday. The city-specific beta-coefficients from Stockholm are also compared with the corresponding results from a meta-analysis [14], presented in Table 2. The reason for the comparisons with the meta-coefficients is to see to what extent they differ from our coefficients obtained in Stockholm.

For the assessment, we use concentrations of NO_x_, O_3_ and PM_10_, based on daily mean values from a central site in Stockholm. The monitoring station is a part of the city’s regulatory air pollution control network and equipped with reference instruments according to the EU air quality directive for NO_x_, O_3_ and PM_10_ (Table 1). It is located on a roof 20 m above the street level and represents urban background concentrations. Air quality dispersion modelling has shown that the urban background concentrations recorded are good indicators of the exposure to the population in the Greater Stockholm area, even though the NO_x_ and PM_10_ exposures are slightly overestimated, while the opposite applies to O_3_ [15]. Birch pollen were sampled in a Burkard trap located on a roof at Stockholm University, about 4 km north of the city center. Daily average number of pollen grains per cubic meter were recorded manually by the Palynological Laboratory at the Swedish Natural History Museum. For birch pollen, the levels are based on measurements during 2015–2017, and the levels are reported as the daily average number of pollen grains per cubic meter.

Local epidemiological data in terms of daily number of AEDV (ICD10: J45-J46) in Greater Stockholm (population 1.2 million) from January 2001 until December 2005 were collected from the National Patient Register at the Swedish National Board of Health and Welfare. The average daily mean of AEDV was 22.2, with a range of 0–71, and with an interquartile range value of 25. Data on daily pollen counts in Stockholm and daily average urban background (roof level) pollutant levels of NO_x_ and PM_10_ were obtained from the same stations as described above. For O_3_, the daily maximum 8-h mean concentration is used. Meteorological observations for Stockholm in terms of temperature and relative humidity were collected from the Swedish Meteorological and Hydrological Institute (SMHI). Data were analyzed by using additive Poisson regression models for over-dispersed counts. The three air pollutants and birch pollen were included as mean of the same day and the day before (lag01), and the meteorological variables as same day (lag 0) and the mean of two previous days (lag12). Time trend and seasonality were adjusted for by using a smooth function (with 3 degrees of freedom per year) using penalized regression splines. Day of week, month and holidays were included with dummy variables, and influenza (according to hospital admissions) was included as a smooth function.

As a validation of the AQHI index, a comparison was made between different regression models determination coefficients (R^2^_adjusted_), generated from inclusion of either AQHI or inclusion of the three air pollutants and birch pollen separately. For this purpose, a new set of data was achieved for the same region: daily counts of AEDV for the time period of 1 January 2005 to 30 November 2013 in Stockholm, Sweden. To clarify, in a first step, a base model was generated with daily counts of AEDV and adjusted for calendar variables, time trend, meteorological data and influenza admissions (as described above but without air pollutants and birch pollen). In a second step, we compared the change of the base models R^2^_adjusted_ values generated when adding the AQHI to the base model, or adding the three air pollutants and birch pollen separately into the base model, for which a higher R^2^_adjusted_ can be interpreted as a better model.

## 3. Results

### 3.1. Validation of the AQHI Index

In our validation analysis of how the AQHI index describes daily observations of AEDV between the years 2005–2013, a higher R^2^_adjusted_ was obtained when including AQHI compared to the inclusion of NO_x_, O_3_, PM_10_ and birch pollen separately. For all age groups (which the AQHI was derived on), an inclusion of AQHI into the regression model (adjusting for calendar variables, meteorological data and influenza admissions) increased the R^2^_adjusted_ from 0.743 to 0.765, which is an increase of 13% higher than when the three air pollutants and birch pollen were included separately.

### 3.2. The Risk-Coefficients that Are Used for the AQHI

In Table 2, the beta-coefficients for AEDV obtained in Stockholm are presented together with the beta-coefficients obtained in a meta-analysis [14]. In the absence of AEDV coefficients for NO_2_ and O_3_ in the meta-analysis, the risk increase of hospital admissions for asthma has been used as a substitute, while AEDV has been used for PM_10_. For Stockholm, the beta-coefficients associated with exposure to PM_10_ and birch pollen are statistically significant, while the coefficients associated with exposure to NO_x_ and O_3_ are not. In the meta-analysis, the coefficients for NO_2_ and PM_10_ are significant, while the coefficient for O_3_ is not. However, all the point estimates representing the average values are positive, and these have been used for further calculations. When comparing the point estimates in Stockholm with the point estimates in the meta-analysis, the coefficients for PM_10_ are reasonable similar, while they are very different for O_3_. The meta-coefficient for NO_2_ is also considerably larger than the NO_x_ coefficient in Stockholm. The studies in the meta-analysis are mainly based on studies from the 90’s, and with many studies from limited parts of the world. Possible reasons for the differences between the meta-coefficients and the coefficients obtained in Stockholm are further analyzed in the discussion section.

### 3.3. The Air Quality Health Index Resulting from the Levels of Air Pollutants and Birch Pollen

**Figure 1:**
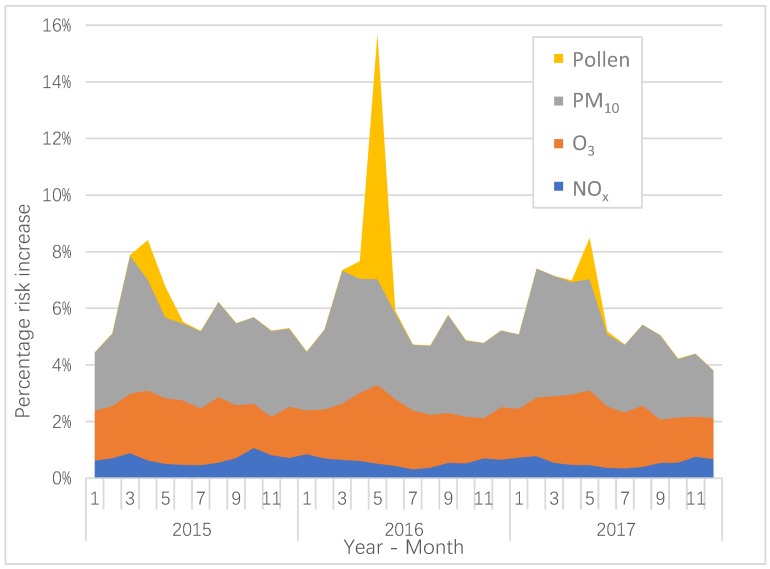
The average monthly AQHI resulting from levels of contributing air pollutants in Stockholm during 2015–2017. The risk coefficients in Stockholm have been used in the calculations.

The monthly average AQHI during 2015–2017, based on NO_x_, O_3_, PM_10_ and birch pollen, has been calculated based on the observed concentrations and risk coefficients from Stockholm (Figure 1). The increase in AQHI resulting from the levels of birch pollen is most prominent during a short period associated with the flowering that occurs during April and May. But the importance of pollen is also quite different for the three years, as shown in Figure 1. The average total monthly risk increase is 6.3% in 2016, while 5.7% and 5.6% are found for 2015 and 2017.

In Table 3, the average AQHI on a daily basis during the period 2015–2017 is calculated for each pollutant. The calculations based on the coefficients in Stockholm are compared with the coefficients based on the meta-analysis [14]. The average daily AQHI based on the meta-coefficients are, especially for O_3_, considerably larger in comparison with the coefficients in Stockholm.

**Figure 2:**
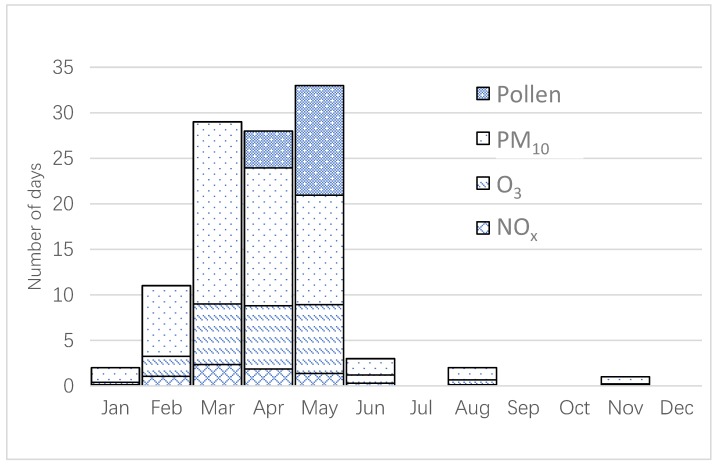
The number of days which exceed the 90th percentile of the risk increase. The calculations are based on the number of monthly exceedances for the whole period from 2015 to 2017.

In Figure 2, the number of days exceeding the AQHI 90th percentile have been calculated on a monthly basis during the period 2015–2017. The number of exceedances are very different during the year, where both March, April and May stand out as there are relatively large numbers of exceedances associated with both O_3_ and PM_10_. April and May stand out with a large number of exceedances for birch pollen, in conjunction with the birch pollen season.

## 4. Discussion

### 4.1. Comparing AQHI and AQI

An AQI based on air quality goals or limit values provides information on the air quality situation in relation to limit values and goals, but it may not be representative of the public health risks associated with air pollution exposure, at least not in a direct way. In Stockholm, the AQI reported by the city’s Environment and Health Administration is based on the daily average concentrations of NO_2_ and PM_10_ obtained from street-level monitoring stations [17]. For PM_10_, the AQI is based on the average concentration from four curbside monitoring sites in central Stockholm. Here should be noted that the measurement data for the calculated AQI differ from the calculated AQHI, as for both NO_x_, O_3_ and PM_10_ are based on measurements from an urban background station. The AQI is “high” when the concentrations exceed the daily limit values, “moderately high” above the upper thresholds but below limit values, “moderate” between the lower threshold and upper threshold and “low” when concentrations are below the lower threshold value. Comparisons between AQI and AQHI in Stockholm show that the information to the public may be very different depending on if it is based on limit values or health risk coefficients (Table 4). Even though the mean AQHI increases as AQI increases, there is a large variation of the AQHI. E.g., for AQI = 3, AQHI varies from 1.7 to 9.5, i.e., from quite small increase in AEDV to a large increase in AEDV.

The AQHI based on the sum of NO_x_, O_3_, PM_10_ and birch pollen shows much larger variability than an AQI based on PM_10_ or NO_2_, and can become high even when AQI’s indicate low concentrations. This variability is especially prominent when the effect of birch pollen is considered.

**Figure A1:**
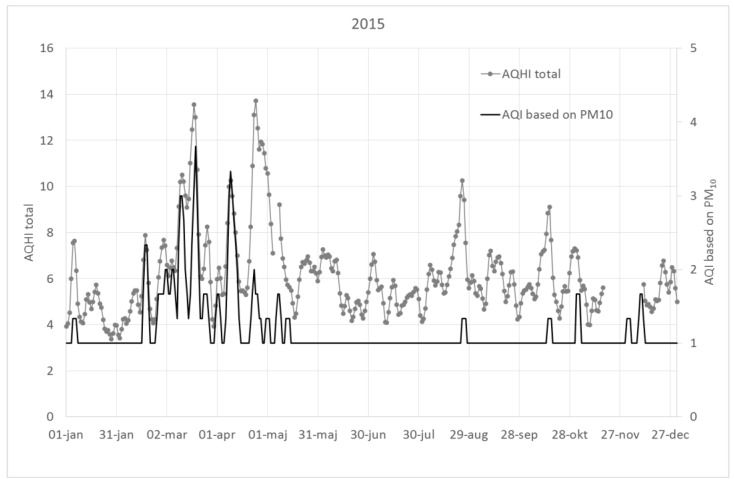
Comparison of 3-day running mean values of AQHI total and AQI PM_10_ in Stockholm during 2015. AQHI is the percentage increase in AEDV associated with combined exposure to NO_x_, O_3_, PM_10_ and birch pollen. AQI is based on a four-point index scale, representing low (PM_10_ ≤ 25 µg·m^–3^), moderate (PM_10_: 25–35 µg·m^–3^), moderately high (PM_10_: 35–50 µg·m^–3^) and high (PM_10_ ≥ 50 µg·m^–3^).

**Figure A2:**
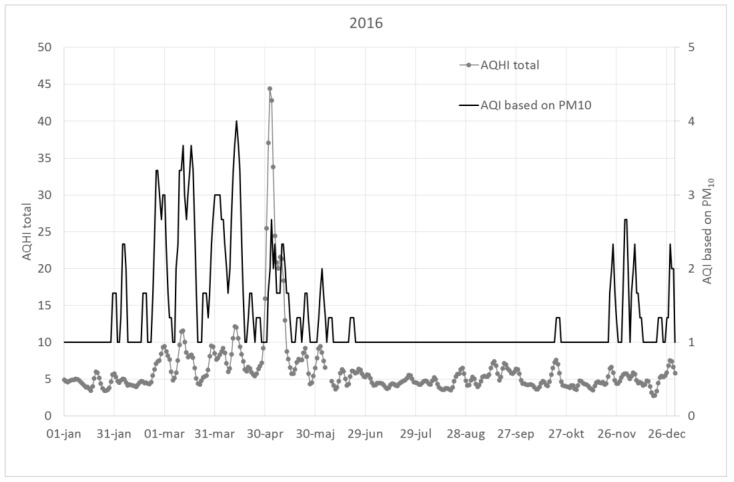
Comparison of 3-day running mean values of AQHI total and AQI PM_10_ in Stockholm during 2016. AQHI is the percentage increase in AEDV associated with combined exposure to NO_x_, O_3_, PM_10_ and birch pollen. AQI is based on a four-point index scale, representing low (PM_10_ ≤ 25 µg·m^–3^), moderate (PM_10_: 25–35 µg·m^–3^), moderately high (PM_10_: 35–50 µg·m^–3^) and high (PM_10_ ≥ 50 µg·m^–3^).

**Figure A3:**
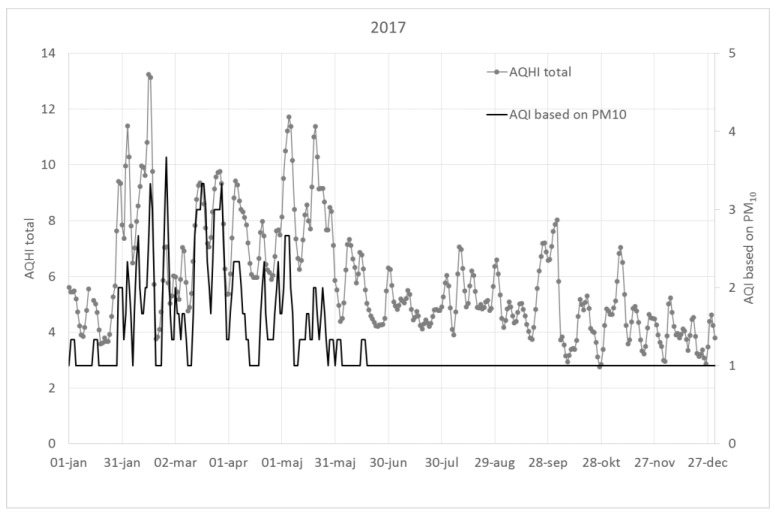
Comparison of 3-day running mean values of AQHI total and AQI PM_10_ in Stockholm during 2017. AQHI is the percentage increase in AEDV associated with combined exposure to NO_x_, O_3_, PM_10_ and birch pollen. AQI is based on a four-point index scale, representing low (PM_10_ ≤ 25 µg·m^–3^), moderate (PM_10_: 25–35 µg·m^–3^), moderately high (PM_10_: 35–50 µg·m^–3^) and high (PM_10_ ≥ 50 µg·m^–3^).

This is illustrated in Figure A1, Figure A2 and Figure A3, which show 3-day running averages of AQHI (sum of all pollutants and birch pollen) as compared to the AQI using only PM_10_ for the years 2015–2017.

### 4.2. Interaction Effects

An AQHI based on the addition of the individual effect of each pollutant may be wrong if there are significant interactions between the pollutants, or if they are indicators of pollutant mixtures coming from the same source (are correlated), and relative risks used for the AQHI come from single pollutant models. There are human experimental studies indicating both synergistic and antagonistic effects associated with multi-pollutant exposures, but the extrapolation of these findings to environmental exposures should be done with care [10]. Most of these studies have used pollutant concentrations that have been significantly higher compared to the corresponding environmental exposures. The evidence of synergism at normally occurring air pollution exposure levels is limited, and in the absence of evidence of synergism or antagonism, the assumption of additive effects may be considered as the default for risk assessments [10]. In other words, despite possible interaction effects, an AQHI based on the sum of the individual effect of each pollutant in multi-pollutant models is the method that has the greatest scientific support. However, since the AQHI presented here also includes birch pollen, it is also a factor to take into account. Simultaneous air pollution exposure has been shown to be able to increase the allergic response associated with pollen exposure [11,12,18,19]. The potential interaction effects are not possible to quantify in the AQHI; this must be done already in the epidemiological studies, but it is important to be aware of such limitations when it comes to adopting additive effects.

### 4.3. The Contribution to the AQHI from the Different Pollutants

We have chosen to include pollutants that are indicators of health effects due to emissions from different sources. NO_x_ is an indicator of vehicle exhaust emissions, and in Stockholm, both diesel and gasoline vehicles contribute to the NO_x_ concentrations [20]. The annual variation of NO_x_ is not as large as for PM_10_ and O_3_ (Figure 1), but it contributes to an increased AQHI during wintertime with more intensive traffic, more stable atmosphere and lower wind speeds. In contrary, during the summer months, the NO_x_ emissions are lower as the traffic intensity decreases, and the meteorological conditions contribute to greater dilution effects. There is also some anti-correlation between NO_x_ and O_3_, which is mainly caused by the net destruction of O_3_ due to the reaction between NO and O_3_.

For O_3_, the highest levels and the highest predicted risk increase during the year arise during April and May, which is shown in Figure 1. Tropospheric ozone concentrations in the Northern Europe maximize during spring, and that is caused by both long-distance transport of global tropospheric ozone, and by a large-scale peak in the local photochemical production [21]. In Sweden, the O_3_ concentrations are lower inside the cities compared to outside due to the titration by NO_x_ [15,22].

The calculated increase in AQHI associated with PM_10_ is most important during late winter and early spring. This is related to the suspension of road dust particles (>1 µm in diameter) that occurs during late winter and spring in Stockholm, when the road surfaces become drier [23,24]. The road dust is due to the use of studded winter tires [25]. As much as 90% of the mass of PM_10_ in Stockholm during the spring originates from road abrasion [23]. Exhaust particles represent only a very small proportion of the total mass of PM_10_ because of their small size. As shown in Figure 1, PM_10_ contributes a large proportion of the total AQHI. Considering the fact that PM_10_ to a large extent is locally generated, local action strategies aimed at reducing the PM_10_ levels may potentially decrease the AQHI.

Birch pollen exhibits a pattern with very high levels during the pollen flowering season that takes place during April to June. As shown in Figure 1, the birch pollen concentrations, and the risk increases associated with those, exhibit a biennial pattern with high concentrations during 2016, but with considerable lower concentrations during 2015 and 2017. This phenomenon is mainly caused by a resource competition between vegetative and reproductive organs of the trees. When the leaves act as a sink of growth substances, this process will also inhibit the development of male inflorescences [26]. An abundant seed production during one year can also reduce the pollen production during the following year [27,28]. However, this biennial pattern is not always distinct, and deviations may sometimes occur [29].

### 4.4. The Difference between the Local Risk Coefficients and the Meta-Coefficients

The difference between the meta-coefficients and the coefficients in Stockholm (Table 2) indicates that there may be real differences between the locations and periods. The meta-coefficients in Anderson et al. [14], where the associations between AEDV and exposure to NO_2_, O_3_ and PM_10_ are presented, are based on studies performed during the 1990’s, and no studies from the Nordic countries are represented. The meta-coefficient for NO_2_ is based on five studies, representing Spain, Hong Kong, Italy, Canada and the UK For O_3_, the meta-coefficient is based on seven studies, representing Spain, Australia, Hong Kong, Italy, the Netherlands, the UK and France. For the meta-coefficient associated with PM_10_ exposure, two out of the four studies have been performed in the U.S., one study in Canada and one in the UK This causes the prevailing conditions in the represented countries to have relatively large impacts on the meta-coefficients. The coefficients from the meta-analysis have been used in order to see if the coefficients obtained in Stockholm are similar.

Despite that NO_x_ is used in Stockholm, while NO_2_ is used in the meta-analysis, the mean value of the coefficient in the meta-analysis is almost three times larger compared to the mean value of the coefficient in Stockholm. NO_x_ and NO_2_ are good indicators of traffic-related exhaust emissions in general, and the use of catalysts and particle filters, which gradually have begun to be used during the 1990’s and until now, may have some significance for this large difference between the coefficients.

For O_3_, the meta-coefficient is considerably larger than the coefficient found in Stockholm. One possible explanation may be that the O_3_ levels tend to by high during the birch pollen season. Considering the fact that only one out of the seven studies in the meta-analysis has made adjustments for pollen exposure may explain the relatively large value of the meta-coefficient, where the pollen exposure can contribute to the risk increase.

For PM_10_, the difference between the coefficients is less compared to the difference for NO_x_/NO_2_ and O_3_. In Stockholm, PM_10_ consists largely on mechanically generated particle from studded tires, which is not the case for most of the studies included in the meta-coefficient. The relatively small difference between these in both cases significant coefficients is therefore surprising. However, this relatively small difference can be a random occurrence.

For birch pollen, the coefficient found for Brussels [12] is three times larger compared to the coefficient found for Stockholm. There are several factors that can affect this difference, which makes it motivated to conduct further studies related to this phenomenon.

All the coefficients in Stockholm are obtained from a multi-pollutant model by adjusting the effect of each individual pollutant from the effects of the other pollutants, which is not the case for the meta-coefficients, and this can of course also contribute to differences between the coefficients. However, when comparing the coefficients from Stockholm with the meta-coefficients, it turns out that the point estimates of the meta-coefficients are within the confidence intervals of the coefficients in Stockholm.

### 4.5. Strengths and Limitations of this Study

An advantage of this study is that the coefficients for the different pollutants are adjusted for each other, which means that there is no risk for double calculations when the risk effects of the different pollutants are added. Another advantage is the inclusion of birch pollen, which have major effects during parts of the year, and where there are possible interaction effects with both exhaust emissions and ozone. The validation of the AQHI, presented in the beginning of in Section 3.1, indicate that the index is a better predictor of AEDV related to air pollution exposure in comparison with the inclusion of each pollutant separately, but where the effect of each pollutant is not adjusted for the effects of the others. As far as we can find, none of the cited studies using an AQHI have tried to validate this index with AEDV by comparing the levels of determination with air pollution data. However, a disadvantage of this study is the less precise local risk coefficients for NO_x_ and O_3_. Follow-up studies, based on coefficients with larger amounts of data, may therefore be beneficial. The air pollution concentrations are based on the measurements from an urban background station, but without considering differences in population exposure, which is also a disadvantage. Another limitation is the use of AEDV, but without considering other more serious outcomes like e.g., mortality.

## 5. Conclusions

By shifting from a threshold-based index (AQI) to a health-risk based index (AQHI), there are several advantages that can be achieved. The cumulative effects arising from combined exposure to several different air pollutants are illustrated by the use of the AQHI. The relative importance of each pollutant during different parts of the year is also illustrated through this multi-pollutant approach. In Stockholm, the risk increase for AEDV associated with combined exposure varies throughout the year, with the largest risk increase during the period from March to June, in conjunction with relatively high levels of PM_10_, O_3_ and birch pollen.

From a policy point of view, there are two particularly important factors that can be achieved by incorporating the AQHI concept in the legislation. The first one is to geographically present the current air quality situation with an index based on the coexistence of several different pollutants. The second one is for forecasting purposes to provide warnings regarding the air quality situation tomorrow or the next few days considering the combined effect of several pollutants. Moreover, public information regarding the health risks associated with different pollutants and pollen can be very useful for sensitive persons when planning their outdoor activities. It may also be important to be able to know if it is pollen or some specific air pollutant that causes the adverse health risks.

## Figures and Tables

**Table 1:** Description of the measurement methods and the instruments that have been used to measure the pollutants included in the AQHI (Air Quality Health Index).

Pollutant	Method	Instrument
**NO_x_**	Chemiluminescence	AC32M, Environnement S.A., Envea, Poissy, France
**O_3_**	UV absorption	O342M, Environnement S.A. Envea, Poissy, France
**PM_10_**	Gravimetric	TEOM 1400A, Thermo Fisher Scientific, Waltham, MA, USA
**Birch pollen**	Manual counting in microscope [16]	Burkard Seven Day Recording Volumetric Spore Trap (Burkard Manufacturing Co. Ltd., Rickmansworth UK)

**Table 2:** The percentage increase in asthma emergency department visits (AEDV) associated with 10 µg·m^–3^ increase of NO_x_, O_3_, and PM_10_ in Stockholm during the period 2001–2005, and from a meta-analysis [14], based on NO_2_, O_3_ and PM_10_. For birch pollen, the percentage increase associated with an increase of ten pollen grains per cubic meter has been used.

Pollutant	Stockholm (Our Analysis)	Meta-Analysis [14]
**NO_x_**	0.5 (95% CI: −1.2–2.2)	Not included
**NO_2_**	Not included	1.4 (95% CI: 0.6–2.2)
**O_3_**	0.3 (95% CI: −1.4–2.0)	1.6 (95% CI: −0.1–3.3)
**PM_10_**	2.5 (95% CI: 0.3–4.8)	3.3 (95% CI: 1.0–5.5)
**Birch pollen**	0.26 (95% CI: 0.18–0.34)	Not included

**Table 3:** The average daily percentage risk increase of each pollutant during the period 2015–2017. The results based on the coefficients in Stockholm are compared with the results based on the meta-coefficients (Table 2). For birch pollen, the risk increase based on the coefficient in Stockholm is compared with the risk increase based on the coefficient obtained in the study from Brussels.

Pollutant	Stockholm	Meta-Analysis [14]	Brussels [12]
**NO_x_/NO_2_**	0.6 (NO_x_)	2.0 (NO_2_)	*
**O_3_**	2.0	10.4	*
**PM_10_**	3.1	4.0	*
**Birch pollen**	0.4	*	1.2
**Sum**	6.1	16.4	1.2

* Not included in the analysis.

**Table 4:** Comparison of AQHI (based on the sum of the risks associated with NO_x_, O_3_, PM_10_ and birch pollen) with AQI for PM_10_ in Stockholm 2015–2017. There are 1021 days with data for all parameters out of the total number of 1096 days.

PM_10_ Index (AQI)	PM_10_ Concentration Intervals (µg·m^−3^) for AQI ^1^	AQHI			Number of Days
		Mean	Min	Max	
1 (low)	<25	2.57	0.52	9	839
2 (moderate)	25–35	4.8	1.9	10.1	95
3 (moderaltely high)	35–50	5.2	1.7	9.5	61
4 (high)	≥50	6.7	2.7	11.6	26

^1^ Index = 1 corresponds to PM_10_ concentrations below the lower assessment threshold, index = 2 corresponds to PM_10_ between the lower assessment threshold and upper assessment threshold, index = 3 corresponds to PM_10_ above the upper assessment threshold, and index = 4 corresponds to PM_10_ above or equal to the EU air quality limit value.
